# Review of Initiatives and Methodologies to Reduce CO_2_ Emissions and Climate Change Effects in Ports

**DOI:** 10.3390/ijerph17113858

**Published:** 2020-05-29

**Authors:** Sahar Azarkamand, Chris Wooldridge, R. M. Darbra

**Affiliations:** 1 Resource Recovery and Environmental Management (R2EM), Department of Chemical Engineering, Universitat Politècnica de Catalunya, BarcelonaTech, Diagonal 647, 08028 Barcelona, Spain; sahar.azarkamand@upc.edu; 2 School of Earth and Ocean Sciences, Cardiff University, Main Building, Park Place, Cardiff CF10 3AT, UK; wooldridge@cardiff.ac.uk

**Keywords:** CO_2_, climate change, carbon footprint, greenhouse gases, ports

## Abstract

Ports are important infrastructures for economic growth and development. Among the most significant environmental aspects of ports that contribute to the issue of climate change are those due to carbon dioxide emissions generated by port activities. Given the importance of this topic, this paper gathers initiatives and methodologies that have been undertaken to calculate and reduce CO_2_ emissions and climate change effects in ports. After studying these methodologies, their strengths and opportunities for further enhancement have been analyzed. The results show that, in recent years, several ports have started to calculate their carbon footprint and report it. However, in some of the cases, not all the sources of GHG gases that are occurring actually in ports are taken into account, such as emissions from waste treatment operations and employees’ commuting. On other occasions, scopes are not defined following standard guidelines. Furthermore, each authority or operator uses its own method to calculate CO_2_ emissions, which makes the comparison of results difficult. For these reasons, this paper suggests the need for creating a standardized tool to calculate carbon footprint in ports, which will make it possible to establish a benchmark and a potential comparison of results among ports.

## 1. Introduction

Ports are important infrastructures for economic growth and development. They have strategic importance to a nation, acting as gateways to trade. They also constitute a key node in the global supply chain [1]. At the same time, they are very complex systems, since each port is unique in terms of activities, geography or applicable laws. Most of them are regulated by diverse levels of legislation: global, European, national and local. 

Apart from generating positive economic development, ports create negative impacts on the environment due to the range and nature of the activities, products and services carried out in the port area. These may have a direct or indirect impact on air, water, soil and sediment, as well as on the quality of life of local communities. Activities such as dredging or the disposal of residues may have negative effects on the movement of water and on the quality of the marine ecosystems, respectively [2]. In general, in ports, almost all the activities can be associated with environmental impacts, such as waste water, emission of gas or particles into the atmosphere, noise, soil contamination, waste production, accidental releases into water or air, etc. [3].

In recent years, several attempts have been made to control environmental impacts in ports. ‘Going green’ is a trend for seaports all over the world, and environmental management has become a critical issue in port operations. The advantages of environmental management are not only for customer satisfaction and corporate image but also for cost saving and environment protection [4].

One of the most important environmental impacts in ports is air pollution [5]. Emissions of exhaust gases and particles from ocean-going ships are a significant and growing contributor to the total emissions from the transportation sector. The intensity of air pollution from fuel combustion depends on the activity of the ship. If the ship is in the open sea, maneuvering, or in the dock, the gases emitted will vary, but they always consist of NO_x_, SO_x_, CO_2_ and suspended particles (PM) [6]. 

In addition, the generation of CO_2_ in this area is one of the significant environmental threats in ports, and this is directly related to climate change [5]. The political significance of this issue has increased in recent years, becoming part of the agenda of numerous international organizations. For instance, according to the International Association of Ports and Harbors [7], growing emissions of greenhouse gases (GHG) have been proved to be the cause of global climate change in port operations. Shipping emissions generate approximately 1036 million tons of GHG emissions annually, and account for 2.4% of global carbon emissions for the periods from 2007 to 2012 [8]. Another example is the results of the Greenport Conference, held in Valencia (Spain) in October 2018, which highlighted the importance of climate change for ports [9]. During this conference, a survey on climate change issues was delivered to the participants, gathering 55 answers from all over the world. From its analysis, it could be stated that climate change occupied the sixth position among the top10 environmental port priorities, and carbon footprint the eighth position. Based on the results of this survey, most of the port organizations (81%) believed that climate change had impacts on their organizations, such as via sea level rise. Most of the respondents of the Greenport survey (86%) considered that GHG emissions from shipping generated in the port area should be included as third-party emission in the carbon footprint calculation of the port. In addition, most of them considered that a common, port-sector carbon footprint scheme would benefit individual port authorities and the port sector as a whole (89%). In 2019, the European Sea Ports organization (ESPO) published its annual environmental review, where climate change occupied the third position among the top 10 environmental priorities [5]. This reflects the importance of climate change and carbon footprint in the whole set of environmental priorities at European and international level.

Climate change impacts, such as the increase in sea level and of storm frequency, will affect seaports and inland waterway infrastructures. As a consequence, due to the economic importance of the ports, their location (in many instances, in the heart of sensitive environments) and the significant existing infrastructure that links them to inland transportation networks, they need special treatment [10]. Therefore, the topic of climate change in the maritime industry is getting more important every day.

It may reasonably be stated that the environmental issues of air quality and sustainability of industrial activities and operations are set to become of even higher priority and significance post Covid-19, given the widely reported improvements in air quality during the “lockdown” period of 2020. Such environmental imperatives will focus further attention on the port sector’s own initiatives if it is to demonstrate competence in the effective management of such critical topics as carbon footprint. It is timely and topical to review the efforts to date, and to research the pathways that will deliver a generic system appropriate to the widely different circumstances of the maritime world.

For this reason, this paper presents a review of different initiatives to reduce climate change effects in general and specifically in the maritime sector. After that, the results of a research conducted on different existing methodologies to calculate CO_2_ emissions in ports are presented, followed by an analysis of their strengths and opportunities for further development. Finally, some conclusions have been drawn. 

## 2. Climate Change and Carbon Footprint Initiatives

Climate change is an important global issue, which has become a major focus of attention because of its potential hazards and impacts on the environment [11]. The on-going global climate change has been related to GHG emissions because of the atmospheric warming effect of these emissions [12]. The main GHGs are carbon dioxide (CO_2_), methane (CH_4_) and nitrous oxide (N_2_O). 

In order to measure the potential contribution of human activities to climate change, an environmental indicator can be used: carbon footprint. Carbon footprint is an environmental indicator that has been developed over the last decade [13,14].

Based on the Parliamentary Office of Science and Technology [15], carbon footprint is the total amount of CO_2_ and other GHG emissions which are emitted over the full life cycle of a process or product. The other GHGs are expressed as CO_2_ equivalent (CO_2_eq). The carbon dioxide equivalent of a quantity of gas is calculated by multiplying the mass of the gas (in tons), by the gas global warming potential (GWP). GWP value for CO_2_ is equal to 1 for a 100-year time horizon, for CH_4_ it is equal to 25 and for N_2_O it is equal to 298 [12]. 

Many international initiatives have been taking place for many years in order to control climate change and carbon footprint. Some of the most significant ones are summarized in Table 1 and explained in more detail after the table.

As can be seen in Table 1, in 1979, the World Meteorological Organization (WMO) sponsored the first major international meeting on climate change in Geneva. In this event, concerns about this topic were expressed and first actions discussed [16].

In 1988, the United Nations Environmental Program (UNEP) and World Meteorological Organization set up the Intergovernmental Panel on Climate Change (IPCC), to provide regular scientific assessments of the current climate change situation and assist policymakers to control it [17]. In addition, IPCC published a set of guidelines for National Greenhouse Gas Inventories in 1995. The revised versions of these guidelines were issued in 2006 and updated in 2019 [18,19].

This was followed in 1992 by the development of the United Nations Framework Convention on Climate Change (UNFCCC) in Rio de Janeiro to stabilize GHG concentrations in the atmosphere at a level that would prevent dangerous anthropogenic interference with the climate system [20].

After this, in 1997, the Kyoto Protocol was adopted in Kyoto (Japan) and entered into force in 2005. This aimed to limit GHG emissions by at least 5% below 1990 levels in the commitment period from 2008 to 2012 [21].

Another interesting attempt is the Global Reporting Initiative (GRI), an international independent organization that has pioneered corporate sustainability reporting since 1997. GRI helps businesses, governments and other organizations understand and communicate the impact of business on critical sustainability issues, such as climate change, human rights, corruption and many others [22].

The development of the Greenhouse Gas Protocol was a very important milestone in the fight against climate change. In 1998, the World Resources Institute (WRI) and the World Business Council for Sustainable Development (WBCSD) developed this protocol. It included standards, tools and online training that helped countries and cities to track progress towards their climate goals [23].

In 1998, the EPA (U.S. Environmental Protection Agency) developed regulations for GHG emissions, such as regulations related to GHG emissions from new motor vehicles and new motor vehicle engines under section 202 of the Clean Air Act [24].

Later on, in 2003, the World Wide Fund for Nature (WWF) and other international NGOs developed the Gold Standard emission allowance. The aim of this project was to ensure that the projects reduced carbon emissions under the UN’s Clean Development Mechanism (CDM) and also contributed to sustainable development. The next generation of this standard launched in 2017, and allowed climate and development initiatives to quantify, certify, and maximize their impacts on climate security and sustainable development [25].

Another important landmark is the development of ISO 14064 by the International Organization for Standard (ISO) in 2006. This international standard includes principles and requirements for designing, developing, managing and reporting organization or company-level GHG inventories [26]. The complete and revised version of this standard was published in 2018 [27].

Bearing in mind the importance of the carbon footprint, in 2007 the Ecological Transition Ministry (MITECO) of the Spanish government developed a tool and guidelines to calculate it. The last version of these guidelines was published in 2019 and they aim to calculate emissions of scope 1 and scope 2 [28].

In the same direction, in 2008, the Catalan Office for Climate Change (Catalonia, Spain) developed an excel-based tool to calculate CO_2_ emissions. The latest version of this tool with its guidelines was published in 2019. The purpose of these guidelines is to facilitate the estimation of GHG emissions [29].

In order to foster training in sustainability issues such as climate change, in 2009, the Partnership for Learning on Climate Change (UN CC: Learn) was launched by the United Nations (UN). The main function of this collaborative initiative was to provide support to countries that wanted to develop and implement training plans in sustainability, addressing in particular climate change [30].

Following the aforementioned Kyoto protocol, in 2015, the Paris Agreement was established. Within the framework of the United Nations Framework Convention on Climate Change, the Paris Agreement recognized climate change as an urgent threat and set the mitigation goal of limiting the global temperature increase up to 2 °C and ideally up to 1.5 °C [31]. However, GHG emissions have continued to rise [32]. 

In 2017, in the UK, the Carbon Trust aimed at developing a common understanding of what the carbon footprint of a product is and circulated a draft methodology for consultation [33]. The Carbon Trust is a private company set up by the UK government to accelerate the transition to a low-carbon economy. The Carbon Trust methodology estimates the total emission of greenhouse gases (GHG) in carbon equivalents from a product across its life cycle, from the production of raw material used in its manufacture to disposal of the finished product (excluding in-use emissions).

The next step after the Paris Agreement was the Conference of Parties (COP 25) of the UNFCCC gathered in Madrid in December 2019. One of the main achievements of this COP was increasing countries’ ambitions to meet the goals of Paris Agreement [34].

Discussions on climate change have thus been evolving at an international scale for around forty years, and the issue remains dynamic in terms of science and politics right up to the current period, with future pathways still to be determined. In the post-covid-19 period, it will surely gain further status in terms of multinational collaboration regarding trans-boundary impacts and the goals of sustainability and overall environmental quality.

In the next section, specific initiatives for climate change and carbon footprint reduction conducted in the maritime sector are presented.

## 3. Initiatives Related to Climate Change and Carbon Footprint in the Maritime Sector

Whereas some GHGs are emitted naturally, there is agreement among climate scientists internationally that human activity has significantly increased the GHGs in the Earth’s atmosphere, leading to accelerating global warming [7]. As is mentioned in the introduction, shipping and port operations are human activities which have an impact on climate change and could be affected by it. Activities causing this warming include those that occur in and around a port, such as burning fossil fuels for operations, transportation, heating and electricity [7].

Several initiatives from international organizations in the maritime sector have been undertaken in the last few years concerning CO_2_ reduction and climate change. These initiatives are summarized in Table 2.

As it can be seen in Table 2, in 1997, Annex VI MARPOL (The International Convention for the Prevention of Pollution from Ships) was adopted by the International Maritime Organization (IMO). The aim of this regulation was to minimize airborne emissions from ships and their contribution to local and global air pollution and environmental problems. Annex VI entered into force in 2005 and a revised Annex VI with significantly tightened emissions limits was adopted in 2008, which entered into force in 2010 [35].

A very important action took place in 2008: the creation of the World Ports Climate Initiative (WPCI). This is a mechanism for assisting ports in controlling climate change, developed by the International Association of Ports and Harbors. The WPCI was developed to reduce the threat of global climate change [7]. In 2010, WPCI developed guidelines to provide a platform for the exchange of information and to improve ports’ GHG emissions inventories [36].

Later on, also at international level, in 2011, IMO adopted a suite of technical and operational measures to provide an energy efficiency framework for ships. These mandatory measures entered into force in 2013, under Annex VI of the International Convention for the Prevention of Pollution from Ships (the MARPOL Convention) [8].

In 2014, PIANC (the World Association for Waterborne Transport Infrastructure) published a Guideline for Port Authorities. This guideline included seven key issues to deal with, and one of them was climate change mitigation and adaptation [37]. More recently, in 2019, PIANC’s Working Group 188 investigated the carbon footprint of activities in navigation channels and port infrastructure, including the management of dredged material [38]. After this, in 2020, PIANC Working Group 178 published a technical guidance document to help the owners, operators and users of waterborne transport infrastructure adapt to climate change [39].

Opening up the calculations of carbon footprints to port stakeholders was one of the objectives of the Clean Cargo Working Group (CCWG). This developed methods to calculate the CO_2_ footprint for a single shipment or a total transportation company. [40].

Concerning ships’ measurements, in 2018 the 72nd session of the Marine Environment Protection Committee (MEPC 72) was held at IMO’s headquarters in London. In this session, the initial IMO strategy on the reduction of GHG emissions from ships was adopted [41].

Another interesting initiative to promote climate change measures among ports was the World Ports Sustainability Program (WPSP). This demonstrated global leadership of ports in contributing to the Sustainable Development Goals of the United Nations, along five themes. The second of these is related to climate change and energy. Based on the output of the WPCI, port community actors can collaborate in developing tools to facilitate the reduction of CO_2_ emissions from shipping, port and landside operations [42].

Reductions of port emissions were also promoted by the World Ports Climate Action Program (WPCAP) launched in 2019 by the world’s biggest ports, including the European ports of Rotterdam, Antwerp, Barcelona and Hamburg [43].

Conferences are also relevant places to gather experts and take decisions. In 2019, the European maritime community met during the Green Ship Technology Conference in Copenhagen, with the aim to reduce GHG emissions from shipping by 50% until 2050 (compared to 2008) based on the IMO decision. They proposed some solutions to reach this goal, such as the implementation of the regulation, compliant fuels and expanding or upgrading existing port infrastructure [44].

In February 2020, ESPO published its position paper on the European Green Deal. According to ESPO, European ports are trying to be the world’s first net-zero-emission area by 2050. By 2030, CO_2_ emissions from ships at berth and in ports should be reduced by 50% on average and across all segments of shipping. In addition, Onshore Power Supply (OPS) should be encouraged as an important part of the solution [45].

Again, it can be seen after all the initiatives presented in this section that the maritime sector has been very active in the last decades trying to establish limits to GHG emissions or creating guidelines to reduce them.

In the next section, research on existing methodologies to calculate CO_2_ emissions and carbon footprint in ports is presented. 

## 4. Research on Existing Methodologies

As mentioned before, in recent years, many ports have started to calculate their carbon footprint and report it. In this paper, the calculation of CO_2_ emissions and carbon footprint in ports, port terminals and ships is studied and analyzed. Ships’ studies are also included since their emissions are contributing to the total port area carbon footprint. More than 20 different methodologies are taken into account. After reviewing all these methodologies, a set of conclusions about their main strengths and opportunities for further enhancement will be extracted in the next section.

### 4.1. Ports

Table 3 presents the ports that were part of this research together with a brief description of their methodologies that will be further explained after the table.

As it can be seen, the Port of Gijón (Spain) was one of the first ports in the world to calculate its carbon footprint (2002), having detected all of the direct and indirect emission sources, which made it possible to establish reduction strategies [46]. In the period from 2004 to 2008, the carbon footprint was calculated again in this port [47]. 

Since 2005, the ports of Long Beach and Los Angeles (San Pedro Bay Ports—SPBP, United States of America) have developed an annual Air Emissions Inventory (EI) report. In November 2006, the ports took joint action to improve air quality in the South Coast Air Basin by adopting the CAAP (Clean Air Action Plan) to ensure that effective air pollution reduction strategies would be commercially available within the five port related source categories: oceangoing vessels, harbor craft, cargo handling equipment, heavy-duty diesel trucks and railroad locomotives [48]. 

Another example is the Port of Oslo (Norway), which calculated its carbon footprint for the first time in 2007 based on ISO 14064-1 by an operational control approach. The results showed that most of the emissions were from fossil fuel combustion (direct source-scope 1), and business travel (scope 3) had the smallest share in the carbon footprint [49].

CLIMEPORT (Mediterranean Ports’ Contribution to Climate Change Mitigation) is a European project that involved six ports committed to climate change mitigation. These ports include the Port Authority of Valencia (Spain), acting as a leader of the project, alongside other port authorities like Algeciras Bay (Spain), Marseille (France), Livorno (Italy), Kopper (Slovenia) and Piraeus (Greece). The objective of this project was to provide a common methodology for port authorities and their collaborators in order to assess their initial situation related to GHG emissions. This methodology provided a way to collect and classify the available information, including questionnaires, invoice data to tenants, and other potential data sources in an ordered way. Concerning vessels, only the captive fleet and oceangoing vessels are considered when berthed in the harbor [50]. The calculation has been done for the year of 2008. In this project, a web-based tool was developed to calculate the carbon footprint of ports. The development of this tool was done using ISO 14064 standards [51]. 

The Port of Rotterdam (The Netherlands) is gradually becoming CO_2_-neutral via the purchase of Gold Standard emission allowances. This is an initiative that was established in 2003 by the World Wide Fund for Nature (WWF) and other international NGOs to ensure that the projects reduce carbon emissions under the UN’s Clean Development Mechanism. The aim of Rotterdam port is to come in line with the Paris Climate Agreement objectives. Port-based companies are encouraged to report their carbon footprint, and the Port of Rotterdam Authority takes steps to reduce its own CO_2_ emissions as well. The Port of Rotterdam Authority is trying to reduce CO_2_ emissions by the use of renewable energy, fuel-saving measures for patrol vessels and electric lease cars for employees [52]. In addition, as the energy consumption and production processes need to switch from fossil fuels to an entirely new system from 2030, a radical transition is needed. A necessary step could be handling energy more efficiently in combination with the capture and underground storage of CO_2_. In this regard, the Port of Rotterdam is also already taking measures to reduce emissions as far as possible in the short term, such as the plan to store CO_2_ below the sea bed in the coming years [53].

Another example is the Port of Stockholm (Sweden), which, since 2012, has reported on sustainability issues according to the GRI (Global Reporting Initiative) in three scopes [54]. The Port of Gothenburg (Sweden) is also working actively to minimize the environmental impact from shipping and to contribute to sustainable transport. Climate and air quality issues are at the top of its agenda. Since 2012, this port calculates the three scopes of carbon footprint and reports them in its annual sustainability report. In 2000, the Port of Gothenburg was the first port to introduce a high-voltage onshore power supply (OPS) for cargo vessels. The implementation of OPS provides an opportunity not only to improve air quality, but also to reduce emissions of carbon dioxide, one of the main contributors to global warming. By switching from fuel oil to gas as an energy source or, better still, to sustainably generated wind power, CO_2_ emissions can be curbed [55].

In 2012, the Port Authority of Barcelona (Spain) joined the voluntary agreements to reduce GHG emissions promoted by the Catalan Climate Change Office (CCCO). By signing this agreement, the port committed to gradually reducing the direct and indirect emissions caused by the fuel consumption of its fleet of 120 vehicles, two boats and certain generators, as well as to reduce its electrical consumption [56]. 

Since 2012, Ports de la Generalitat (Catalonia, Spain) has joined the voluntary agreements program for the reduction of GHG emissions. In this regard, they started to calculate GHG emissions every year by the use of the tool which was developed by the Catalan Office for Climate Change (OCCC) [57].

The Climate Action Plan (CAP) was developed by the San Diego Unified Port District in 2013 (United States of America) to identify, assess and develop strategies to reduce GHG emissions [58].

The carbon footprint of the Port of Chennai (India) was estimated for the year 2014–2015 based on the WPCI guidelines. Misra et al. [59] elaborated an inventory of GHG emissions for the Port of Chennai (India), accounting for the various facilities of the port along with the housing colony and fishing harbor, which come under the management of the Port of Chennai.

In 2007, the Port Authority of Ferrol-San Cibrao (Spain) implemented its environmental sustainability plan. In 2016, the Ferrol-San Cibrao Port Authority started to monitor its environmental aspects through the Integrated Quality and Environmental Management System. Within this frame, GHG emissions in scope 1 and 2 were calculated by the use of the Ecological Transition Ministry (MITECO) tool of the Spanish government [60].

In 2016, the carbon footprint report for operational activities of Giurgiulesti International Free Port (Moldavia) on an annual basis was developed [61]. The GHG Protocol is used to prepare the carbon footprint report [23]. 

In 2016, the Port of Taichung (Taiwan) created a GHG emissions management and reduction plan by a self-management method. This was approved by the Environmental Protection Bureau (EPB) of Taichung City. The approach included an inventory and actions to reduce carbon levels and air pollutants. In this regard, the GHG inventory tool (based on ISO 14064) was developed by the Industrial Development Bureau (IDB) at the Ministry of Economic Affairs and Environmental Protection Agency (EPA) of Taiwan. Following the successful experience of Taichung Port, the self-management method was adopted in other industries and areas in Taichung City [62].

Finally, the Port of Olympia (United States of America), as part of its commitment to environmental sustainability, is voluntarily conducting biennial greenhouse gas emissions inventories. The Washington State Agencies GHG calculator was used to perform the GHG emissions inventory for the port. Scope 1 and Scope 2 emissions were calculated for the 2017 inventory. The use of this methodology facilitates in-state comparison and better helps to demonstrate the Port of Olympia’s contribution to the State of Washington’s overall GHG emissions [63].

### 4.2. Port Terminals

Besides the studies in ports, research on the methods used to calculate CO_2_ emissions and carbon footprint in port terminals has also been conducted. As it can be seen in Table 4, the amount of initiatives is not as extensive as in ports.

Another study from van Duin and Greelings [64] provides insight into the processes of container handling and transshipment at the terminals and calculates the contribution of these processes to the carbon footprint of the container terminals in Netherlands. An activity-based emission modeling was applied to develop a methodology for the calculation of emissions caused by the container terminals.

In research from Chowhan et al. [65], the three scopes of emission in four container terminals in two ports in Mumbai were analyzed using the formulae in a spreadsheet developed especially for the computation of carbon footprint based on IPCC guidelines [18]. In this research, a study on the damage of CO_2_ emissions to human health was also carried out. Although the emissions from ships are calculated in Chowhan’ research, the method is not explained.

Finally, in research from Yang [66], carbon footprint analysis was employed to calculate the CO_2_ emissions per container of two different container terminals in the Port of Kaohsiung (Taiwan). The total energy consumption of each type of equipment was calculated as the total working time of that equipment multiplied by the equipment’s energy consumption per hour. The average energy consumption of the equipment was calculated as the equipment’s total energy consumption divided by the quantity of the equipment. Finally, the CO_2_ emissions of each piece of equipment were obtained from the average energy consumption for that piece of equipment multiplied by the CO_2_ emission coefficient.

### 4.3. Ships

As for the port terminals, the initiatives related to the calculation of carbon footprint in ships is reduced. Table 5 presents existing methodologies related to ships.

A study from Chang et al. [67] measured GHG emissions from port vessel operations by considering the case of Korea’s Port of Incheon. It provided an estimation of GHG emissions based on the type and the movement of a vessel from the moment of its arrival to its docking, cargo handling, and departure. 

In a study by Winnes et al. [68], the potential reductions of ships’ GHG emissions due to the implementation of different measures by ports were quantified. This research presents a case study of ship traffic in the Port of Gothenburg in 2010. A case study of the ship traffic at the Port of Gothenburg was performed. Projections of ship emissions in the port area for 2030 with three scenarios were made: alternative fuel use, ship design and operational measures.

The Port of London Authority (PLA) and Transport for London (TfL) requested that Aether. A company that provides consultancy in air quality and climate change emissions inventories, forecasting and policy analysis and TNO Netherlands Organization for Applied Scientific Research on applied science) prepare an inventory of air emissions from shipping on the Thames and other navigable waterways in the Port of London. This inventory provided a baseline against which policy scenarios can be tested to show their impact on pollution emissions along the Thames. The methodology for this study used detailed data on ships and their movements [69]. 

Olukanni and Esu [70] estimated the amount of GHGs emitted from port vessel operations in the Lagos and Tin Can ports of Nigeria. The emission estimate was carried out based on the type of vessel and its movement from the moment of its arrival. The emission estimate was done using the bottom-up approach based on the characteristics of individual vessels and using data on vessels processed by both ports in the first and second quarters of the year 2017. 

## 5. Strengths and Opportunities for Further Development

As it can be seen in the previous section, in recent years, many ports have started to calculate their carbon footprint and report it. However, each port uses its own method and there is no single or unified method to calculate carbon footprint in ports. More than 20 different methodologies used by 15 ports, 3 port terminals and 4 ships were taken into account.

A deep analysis was conducted to study the strengths and opportunities for further development of each methodology that has been presented previously. The percentages have been calculated based on the existence of those strengths or opportunities for further development in the studied cases.

**Figure 1:**
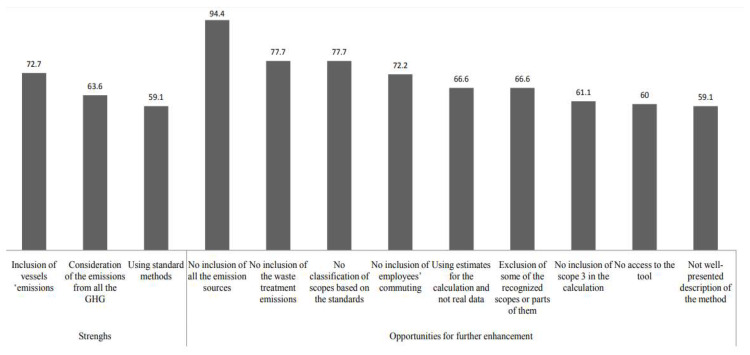
Percentage of the strengths and opportunities for further enhancement of the existing methodologies.

After reviewing all these methodologies, a set of conclusions about their main strengths and opportunities for further enhancement were extracted. They can be seen in Figure 1.

As it can be seen in Figure 1, the main strengths of these studies are:

- In most of the methodologies, vessels’ emissions are taken into account. Taking into consideration the fact that, in 2012, GHG emissions from international shipping already represented already 2.2% of total CO_2_ emissions [8] and that it is also known that such emissions could grow by between 50% and 250% by 2050, it is a very important sign of the awareness of the port sector to include the calculation of emissions from waterborne vehicles in the existing methodologies.
- In around 60% of the methods, not only CO_2_ emissions are calculated, but also other GHG emissions are taken into account such as CH_4_ and N_2_O. This is very important, since, as mentioned previously in Section 2, the warming potential of CH_4_ and N_2_O is much higher than that of CO_2_. Therefore, it is really important to take into account all the gases in the carbon footprint calculation to obtain a real estimate.
- In more than half of the cases, the calculation has been done based on standard methods such as the GHG protocol, IPCC, WPCI and ISO 14064. This makes the calculation more reliable and standard since all these methods should include the same parameters.

As it can be seen in Figure 1, the analyzed methods present scope for further development:

- In almost all of the studies, all the emission sources mentioned in the standard guidelines (direct or indirect) are not calculated. For example, on some occasions, some sources, like emissions from construction equipment or emissions resulting from energy use in rented out buildings are not calculated. In order to obtain comprehensive and realistic figures on GHG emissions and carbon footprint in ports, all emission sources should be taken into account.
- In most of the cases, emissions from waste operations that can take place in a port such as incinerators or wastewater treatment plants are not included in the calculation. This is an opportunity for further enhancement of the existing methods. These emissions should be taken into account, where they exist, since they are sources of CO_2_ emissions that should be counted in the total carbon footprint of a port.
- In most of the studies, scopes are not defined based on the standard methods. For example, in one port, scopes are divided into inside port emissions, outside port emissions and other emissions. In another method, water consumption is calculated in scope 3.
- In around 70% of the cases, emissions from employees’ commuting are not included. These are a very important source of emissions in scope 3. Therefore, their inclusion could help the existing methods to obtain more realistic results of the carbon footprint.
- In those studies, in which information was available, it has been seen that, in general, estimates are used for the calculation and not real data.
- In around 65% of cases, some of the recognized scopes or parts of them are excluded. For example, the calculation of the scope 3 emissions is not taken into account in some ports or scope 2 is excluded from the total GHG calculation in others. To obtain a real figure of carbon footprint in ports, it is recommended to calculate emissions of the three scopes.
- In around 60% of the studies, the whole set of scope 3 emissions (i.e., emissions from tenants, vessels and employees’ commuting) are not calculated. Therefore, the total amount of CO_2_ emissions would not present a real figure for the carbon footprint in that particular port.
- In about 60%of the studies where a tool has been developed (five cases), access to this tool is not possible.
- In more than half of the studies, the methodology is not fully described. Therefore, it is not possible to reproduce it. This could be easily solved, and, in this way, other port agents could use it.

## 6. Conclusions

Ports are strategic nodes for a country’s economy. However, given the range of activities, products and services associated with their operations (including those of their tenants), they also generate impacts on the environment which need to be controlled or minimized by effective environmental management programs in order to achieve compliance and sustainability. One of the significant environmental challenges for ports nowadays is to manage their contribution to climate change, mainly due to emissions from mobile sources and stationary sources in ports. Emissions from these sources, such as emissions from ships, trucks, cargo handling equipment, power plants and others, are the main cause of climate change in ports. As mentioned before, based on the ESPO’s 2019 survey, climate change occupies the third position among the top 10 environmental priorities [5]. This reflects the importance of climate change in the whole set of environmental priorities at European and international level.

In this paper, initiatives to reduce the effects of climate change, carbon footprint and CO_2_ emissions in ports have been studied to identify function and applicability. The results of this research confirm that a range of international organizations and ports are implementing measures to fight against climate change effects and to reduce CO_2_ emissions. 

International organizations such as IMO, IAPH and PIANC have demonstrated commitments to reduce the GHG emissions in ports through different initiatives. Examples of this are the revision of the MARPOL Annex VI to include regulations for the prevention of air pollution from ships, the creation of a mechanism to assist ports in the mitigation of climate change and the development of the World Ports Climate Initiative (WPCI) working group. 

The port sector has also decided to combat climate change with initiatives from several individual authorities. The first step was to calculate their carbon footprint. Ports such as Gijón (Spain) and Oslo (Norway) were the first ones to do so. In recent years, more evolved initiatives consisting of CO_2_ calculators have also been implemented, such as the Washington State Agency GHG calculator.

Concerning the control of CO_2_ emissions in port terminals, some attempts have been made. For example, the three scopes of emission in four container terminals in two ports in Mumbai were analyzed. In some cases, carbon footprint analysis was employed to calculate the CO_2_ emissions per container, such as two different container terminals at the Port of Kaohsiung in Taiwan. 

Finally, ships’ initiatives to reduce GHG gases have also been presented. This includes the case of the study of the ship traffic in the Port of Gothenburg to quantify potential reductions of ship GHG emissions.

The review of different studies shows that, in recent years, many ports have started to calculate their carbon footprint and report it. This is a positive sign in terms of the “greening” of ports. However, there is scope for further enhancement. For example, in most of the cases, all the emission sources mentioned in the standard guidelines (direct or indirect) are not calculated, and emissions from waste treatment operations and employees’ commuting are excluded from the total calculation of CO_2_. This makes the current calculations in some occasions unrealistic.

In addition, in more than half of the studies, no scopes recommended by the standard guidelines are considered and data estimates are used for the calculation and not real data. Additionally, the involvement of the port terminals in the final calculation is lower than expected and this is essential to obtain a real value of emissions for the whole port areas. Their emissions should be added to the final calculation, although it is complicated to attain this data. An effort in these respects could provide more reliable results.

As a general comment, each port uses its own method, and this does not allow establishing a sector benchmark or comparing the results between different ports. There is thus no single or unified method to calculate carbon footprint in ports. All this proves the need for the development a standardized methodology for CO_2_ calculation in ports. ESPO’s declared environmental policy of compliance through voluntary self-regulation needs a practicable and effective methodology by which to monitor and thereby reduce CO_2_ emissions. The initiatives flagged in this review demonstrate commitment by the sector but also highlight the challenges of developing and implementing a generic system that may be applicable throughout the sector, as many observers agree that “each port is unique” (in terms of key characteristics including activity profile and geography). Analysis of the methods available to date highlights the need for a collaborative approach.

This was supported by most of the participants in Greenport Congress in Valencia, as mentioned previously, who consider that a common port-sector carbon footprint scheme would benefit individual port authorities and the port sector as a whole. This is also the view of some of the aforementioned organizations (such as Laboratorio de ingeniería sostenible), which also recognize the need for such a tool. Therefore, the development of a practicable, user-friendly and easy-to-use tool with a standardized method for the calculation of carbon footprint in ports is highly recommended. This tool should include all the strengths of the existing methods and the opportunities highlighted in this paper for further development.

## Figures and Tables

**Table 1:** Summary of international initiatives to control climate change and carbon footprint in general.

Year	Organization	Significance of Initiatives
1979	World Meteorological Organization (WMO)	This was one of the first major international meetings on climate change.
1988	United Nations Environmental Program (UNEP) and World Meteorological Organization (WMO)	This set up the Intergovernmental Panel on Climate Change (IPCC) to provide policymakers with regular scientific assessments on the current state of knowledge about climate change.
1992	United Nations (UN)	The UN developed the United Nations Framework Convention on Climate Change (UNFCCC) to stabilize GHG concentrations in the atmosphere.
1995	Intergovernmental Panel on Climate Change (IPCC)	This published a set of guidelines for national GHG inventories. The revised versions of these guidelines were issued in 2006 and updated in 2019.
1997	United Nations (UN)	The UN developed the Kyoto Protocol, which established an action to limit GHG emissions by at least 5% below 1990 levels in the commitment period from 2008 to 2012.
1997	GRI (Global Reporting Initiative)	The GRI helps businesses, governments and other organizations to understand and communicate the impact of business on critical sustainability issues such as climate change.
1998	World Resources Institute (WRI) and World Business Council for Sustainable Development (WBCSD)	This developed the GHG protocol in order to establish frameworks to measure and manage GHG emissions from private and public sector operations, value chains and mitigation actions.
1998	U.S. Environmental Protection Agency (EPA)	The EPA prepared a legal opinion concluding that CO_2_ emissions were within the scope of the EPA’s authority to regulate.
2003	World Wide Fund for Nature (WWF)	The WWF established the Gold Standard emission allowance to ensure that the projects reduced carbon emissions under the UN’s Clean Development Mechanism (CDM).
2006	International Organization for Standardization (ISO)	The ISO developed ISO 14064, which contains detailed principles and requirements for designing, developing, managing and reporting organization or company level GHG inventories. The revised version of this standard was developed in 2018.
2007	Ecological Transition Ministry (MITECO) of the Spanish government	This developed a tool and a guideline to calculate carbon footprint for scope 1 and scope 2.
2008	Catalan Office for Climate Change (OCCC)	This developed an excel-based tool to calculate CO_2_ emissions in three scopes. The latest version of this tool with its guidelines was published in 2019.
2009	United Nations (UN)	The UN launched the Partnership for Learning on Climate Change.
2015	United Nations (UN)	The Paris Agreement set the mitigation goal of limiting the global temperature increase to 2 °C and ideally to 1.5 °C.
2017	World Wide Fund for Nature (WWF)	The WWF established a next generation of the Gold Standard to quantify, certify, and maximize impacts on climate security and sustainable development.
2017	Carbon Trust (UK based company)	It introduces two types of carbon footprinting that affect businesses: one that measures an organization’s overall activities, and one that looks at the life cycle of a product or service.
2019	United Nations (UN)	The main aim of COP 25 in Madrid is increasing countries’ ambitions to meet the goals of the Paris Agreement.

**Table 2:** Summary of international initiatives to control climate change and carbon footprint in the maritime sector.

Year	Organization	Initiatives’ Explanation
2005	The International Maritime Organization (IMO)	This established regulations for the prevention of air pollution from ships in 1997, and the addition of Annex VI to MARPOL entered into force in 2005.
2008	International Association of Ports and Harbours (IAPH)	This provided a mechanism for assisting the ports in mitigating climate change. It also developed the World Ports Climate Initiative (WPCI), established to raise awareness in the port and maritime community concerning the need for action regarding GHG emissions.
2010	World Ports Climate Initiative (WPCI)	This developed guidelines for ports to create or improve their GHG emissions inventories.
2011	The International Maritime Organization (IMO)	This provided an energy efficiency framework for ships.
2014	The World Association for Waterborne Transport Infrastructure (PIANC)	This published a guideline for port authorities to create awareness about the green port philosophy.
2015	The Clean Cargo Working Group (CCWG)	This developed tools to calculate the CO_2_ footprint for a single or whole approach in the logistic chain.
2018	Marine Environment Protection Committee (MEPC 72)	This adopted IMO strategy on reduction of GHG ship emissions.
2018	The World Ports Sustainability Program (WPSP)	This committed to demonstrating leadership of ports in CO_2_ reduction through the subscription of ports to the Paris Agreement.
2019	PIANC’s Working Group 188	This investigated the carbon footprint of activities in navigation channels and port infrastructure, including the management of dredged material.
2019	World Ports Climate Action Program (WPCAP)	This facilitates emissions reductions from the ports’ supply chains and their larger geographical area.
2019	The Green Ship Technology Conference	This adopted the IMO strategy to reduce GHG emissions from shipping by 50% until 2050.
2020	PIANC Working Group 178	This prepared a technical guidance document to help waterborne transport to adapt to climate change.
2020	European Sea Ports Organization (ESPO)	This published a position paper concerning the European Green Deal, in whichCO_2_ emissions from ships at berth and in ports should be reduced by 50% on average and across all segments of shipping by 2030.

**Table 3:** Existing methodologies to calculate carbon footprint in Ports.

Name of the Port	Description	Year
The Port of Gijón	This was one of the first ports that calculated its carbon footprint (including direct/indirect emissions).	2002
The Ports of Long Beach and Los Angeles	This developed an annual air emissions inventory report. The ports took joint action to improve air quality in the South Coast Air Basin by adopting the CAAP (Clean Air Action Plan).	2005
The Port of Oslo	This calculated the carbon footprint for all the operations under its control.	2007
CLIMEPORT	This provided a common methodology to assess the initial situation of partner ports concerning GHG emissions.	2008
The Port of Rotterdam	This has been CO_2_-neutral since 2011 and encourages port-based companies to report their carbon footprint.	2011
The Port of Stockholm	This has reported sustainability issues according to the GRI.	2012
The Port of Gothenburg	This calculates the three scopes of carbon footprint and reports them in its annual sustainability report.	2012
The Port of Barcelona	This joined the voluntary agreements to reduce GHG emissions promoted by the Catalan Climate Change Office.	2012
Ports de la Generalitat	This started to calculate GHG emissions every year using the tool developed by the Catalan Office for Climate Change (OCCC).	2012
The Port of San Diego	This developed the Climate Adaptation Plan (CAP).	2013
The Port of Chennai	This quantified GHG emissions following the WPCI guideline.	2014
The Port Authority of Ferrol-San Cibrao	This calculated scope 1 and scope 2 GHG emissions by the use of Ecological Transition Ministry (MITECO) tool of the Spanish government.	2016
Giurgiulesti International Free Port	This reported its operational activities on carbon footprint according to the GHG protocol.	2016
Taichung Port (Taiwan)	This established a self-management approach to control the total quantity of GHG from various sources in the port district.	2016
The Port of Olympia (USA)	This conducted voluntary biennial GHG emissions inventories using the Washington State Agencies GHG calculator.	2017

**Table 4:** Existing methodologies in port terminals.

Name of the Port	Description	Year
The Netherlands	This calculated the contribution of the processes of container handling and transshipment to the carbon footprint.	2011
Mumbai—India	This analyzed the three scopes of emission in four container terminals including an assessment of its damage to human health.	2012
Taiwan	This calculated the CO_2_ emissions per container of two different container terminals.	2017

**Table 5:** Existing methodologies related to ships.

Name of the Country	Description	Year
Korea	Estimated the GHG emissions from port vessel operations.	2013
Gothenburg	Implemented measures to reduce GHG ship emissions based on alternative fuel use, ship design and operational measures.	2015
England	Prepared an inventory of air emissions from shipping on the Thames and other navigable waterways in the Port of London.	2017
Nigeria	Estimated the amounts of GHG from port vessel operations.	2017

## References

[1] Wright P. (2013). Impacts of climate change on ports and shipping. MCCIP Sci. Rev..

[2] Gularte Quintana C., Munhoz Olea P., Raggiabdallah P., Costa Quintana A. (2016). Port environmental management: Innovations in a Brazilian public port. RAI Rev. Adm. Inov..

[3] Darbra R.M., Ronza A., Casal J., Stojanovic T.A., Wooldridge C. (2004). The Self Diagnosis Method. A new methodology to assess environmental management in sea ports. Mar. Pollut. Bull..

[4] Teerawattana R., Yang Y. (2019). Environmental Performance Indicators for Green Port Policy Evaluation: Case Study of Laem Chabang Port. Asian J. Shipp. Logist..

[5] ESPO (European Sea Ports Organisation) (2019). Environmnetal Report 2019 EcoPorts in Sights 2019.

[6] Eyring V., IsaksenI S.A., Berntsen T., Collins W.J., CorbettJ J., Endresen O., Grainger R.G., Moldanova J., Schlager H., Stevenson D.S. (2009). Transport impacts on atmosphere and climate: Shipping. Atmos. Environ..

[7] IAPH (International Association of Ports and Harbors) (2010). IAPH Tool Box for Port Clean Air Programs. http://iaphtoolbox.wpci.nl/DRAFTIAPHTOOLBOXdea.pdf.

[8] IMO (International Maritime Organization) (2014). Third IMO Greenhouse Gas Study 2014. http://www.imo.org/en/OurWork/Environment/PollutionPrevention/AirPollution/Documents/ThirdGreenhouseGasStudy/GHG3ExecutiveSummaryandReport.pdf.

[9] Greenport (2019). Balancing Environmental Challenges with Economics Demands. Reports on an Emissions Survey Undertaken at GreenPort Congress. https://issuu.com/mercatormedia/docs/_greenport_summer_2019_flipbook.

[10] Becker A., Inoue S., Fischer M. (2011). Climate Change Impacts and Adaptation: Knowledge, perceptions, and planning efforts among port administrators. Climatic. Chang..

[11] Sánchez-Arcilla A., Mösso C., Sierra J.P., Mestres M., Harzallah A., Senouci M., Raey M. (2011). Climatic Drivers of Potential Hazards in Mediterranean Coasts. Environ. Chang..

[12] IPCC (Intergovernmental Panel on Climate Change) (2008). Climate Change Synthesis Report (AR4). https://www.ipcc.ch/site/assets/uploads/2018/02/ar4_syr_full_report.pdf.

[13] Peters G.P. (2010). Carbon footprints and embodied carbon at multiple scales. Curr. Opin. Environ. Sustain..

[14] Wiedmann T., Minx J. (2008). A Definition of ‘Carbon Footprint’. Ecological Economics Research Trends.

[15] POST (2006). Carbon Footprint of Electricity Generation. https://www.parliament.uk/documents/post/postpn268.pdf.

[16] Sprinz D., Luterbacher U. (1996). International Relations and Global Climate Change.

[17] IPCC (Intergovernmental Panel on Climate Change) (2015). IPCC Factsheet: Timeline—Highlights of IPCC History. https://www.ipcc.ch/site/assets/uploads/2018/04/FS_timeline.pdf.

[18] IPCC (Intergovernmental Panel on Climate Change) (2006). IPCC Guidelines for National Greenhouse Gas Inventories. https://www.ipcc-nggip.iges.or.jp/public/2006gl/pdf/2006gls_all_in.zip.

[19] IPCC (Intergovernmental Panel on Climate Change) (2019). 2019 Refinement to the 2006 IPCC Guidelines for National Greenhouse Gas Inventories. https://www.ipcc-nggip.iges.or.jp/public/2019rf/pdf/2019rf_all_in.zip.

[20] UNFCC (United Nations Framework Convention on Climate Change) (1992). United Nations Framework Convention. https://unfccc.int/resource/docs/convkp/conveng.pdf.

[21] United Nations (1998). Kyoto Protocol, Review of European Community and International Environmental Law. https://unfccc.int/resource/docs/convkp/kpeng.pdf.

[22] GRI (Global Reporting Initiatives) (2019). A Closer Look at Water and GHG Emissions Disclosure Mapping Reporting Practice to an Investor Perspective. https://www.globalreporting.org/resourcelibrary/A-Closer-Look-At-Water-and-GHG-Emissions-Disclosure.pdf.

[23] WRI and WBSCD (World Resources Institute and World Business Council for Sustainable Development) (2004). GHG Protocol. https://ghgprotocol.org/sites/default/files/standards/ghg-protocol-revised.pdf.

[24] NACCA (National Association of Clean Air Agencies) (2013). Background and History of EPA Regulation of Greenhouse Gas (GHG) Emissions Under the Clean Air Act & National Association of Clean Air Agencies’ Comments on EPA GHG Regulatory and Policy Proposals. http://4cleanair.org/Documents/Background_and_History_EPA_Regulation_GHGs-Aug2013-post.pdf.

[25] Ecofys (2006). The Gold Standard, Voluntary Emission Reductions. https://www.goldstandard.org/sites/default/files/documents/developermanual_gs-ver_v.1.pdf.

[26] ISO (International Organization for Standard) (2006). ISO 14064-1 First Edition, Greenhouse Gases, Part1 Specification with Guidance at the Organization Level for Quantification and Reporting of Greenhouse Gas Emissions and Removals. https://www.iso.org/obp/ui/#iso:std:iso:14064:-1:ed-1:v1:en.

[27] ISO (International Organization for Standard) (2018). ISO 14064-1 Second Edition, Greenhouse Gases, Part1 Specification with Guidance at the Organization Level for Quantification and Reporting of Greenhouse Gas Emissions and Removals. https://www.iso.org/obp/ui/#iso:std:iso:14064:-1:ed-2:v1:en.

[28] MITECO (Ministerio para la Transición Ecológica) (2019). Calculadora de Huella de Carbono de una Organización. Alcance 1+2. https://www.miteco.gob.es/es/cambio-climatico/temas/mitigacion-politicas-y-medidas/calculadoras.aspx.

[29] OCCC (Catalan Office for Climate Change) (2019). Practical Guide for Calculate Greenhouse Gas (GHG) Emissions. https://canviclimatic.gencat.cat/web/.content/04_ACTUA/Com_calcular_emissions_GEH/guia_de_calcul_demissions_de_co2/190301_Practical-guide-calculating-GHG-emissions_OCCC.pdf.

[30] United Nations Institute for Training and Research (2015). Resource Guide for Advanced Learning on the Scientific Fundamentals of Climate Change. https://www.uncclearn.org/sites/default/files/guide_scientific_fundamentals.pdf.

[31] (2015). United Nations, Paris Agreement, Adoption of the Paris Agreement. https://unfccc.int/sites/default/files/english_paris_agreement.pdf.

[32] Le Quéré C., Andrew R.M., Canadell J.G., Sitch S., Korsbakken J.I., Peters G.P., Manning A., Boden T.A., Tans P.P., Houghton R.A. (2016). Global Carbon Budget. Earth Syst. Sci. Data.

[33] Carbon Trust (2017). Carbon Footprinting. https://www.carbontrust.com/resources/carbon-footprinting-guide.

[34] (2019). El National, Les Deuclaus de L’acord de la Cimera del Clima de Madrid. https://www.elnacional.cat/ca/societat/claus-acords-cimera-clima-2019_451737_102.html.

[35] IMO (International Maritime Organization) (2019). Air Pollution, Energy Efficiency and Greenhouse Gas Emissions. http://www.imo.org/en/OurWork/Environment/PollutionPrevention/AirPollution/Pages/Default.aspx.

[36] (2010). WPCI (World Ports Climate Initiative). Carbon Footprinting for Ports, Guidance.

[37] PIANC (World Association for Waterborne Transport Infrastructure) (2014). Sustainable Ports’A Guide for Port Authorities. https://sustainableworldports.org/wp-content/uploads/EnviCom-WG-150-FINAL-VERSION.pdf.

[38] PIANC (World Association for Waterborne Transport Infrastructure) (2019). EnviCom 188: Carbon Management for Port and Navigation Infrastructure. J. Chem. Inf. Model..

[39] PIANC (World Association for Waterborne Transport Infrastructure) (2020). Climate Change Adaptation Planning for Ports and Inland Waterways. https://www.pianc.org/uploads/publications/reports/WG-178.pdf.

[40] CCWG (Clean Cargo Working Group) (2015). Clean Cargo Working Group Carbon Emissions Accounting Methodology. https://www.bsr.org/reports/BSR_CCWG_Carbon_Emissions_Methodology_2015.pdf.

[41] IMO (International Maritime Organization) (2018). Initial IMO strategy on Reduction of GHG Emissions from Ships (IMO). http://www.imo.org/en/OurWork/Environment/PollutionPrevention/AirPollution/Documents/ResolutionMEPC.304(72)E.pdf.

[42] Wpsp (World Ports Sustainability Program) (2018). World Ports Sustainability Program. https://sustainableworldports.org/wp-content/uploads/wpsp-declaration.pdf.

[43] Greenport (2019). Cooperation on Climate Action. https://www.greenport.com/news101/europe/cooperation-on-climate-action.

[44] BPO (2019). Collaboration Maritime Industry’s Path to 2030. http://www.bpoports.com/collaboration-maritime-industry’s-path-to-2030.html.

[45] ESPO (European Sea Ports Organisation) (2020). ESPO’s Roadmap to Implement the European Green Deal Objectives in Ports. https://www.espo.be/media/ESPOGreenDealpositionpaperGreenDeal-FINAL.pdf.

[46] Laboratorio de Ingeniería Sostenible (2004). 9 Years of Carbon Footprint in a Spanish Port. http://www.lis.edu.es/uploads/06a2431c_85a6_4952_a7da_f4a90e3309b5.pdf.

[47] Carballo-penela A., Mateo-mantecón I., Doménech J.L. (2012). From the motorways of the sea to the green corridors’ carbon footprint: The case of a port in Spain. J. Environ. Plan. Manag..

[48] (2016). Ports of Long Beach and Los Angeles, Clean Air Action Plan Technology Advancement Program. http://www.cleanairactionplan.org/wp-content/uploads/2016/11/CAAP-2017-Draft-Discussion-Document-FINAL.pdf.

[49] (2008). Port of Oslo, CO_2_ Emissions for the Calendar Year 2008. http://www.renoslofjord.no/sfiles/52/50/5/file/co2-footprint-port-of-oslo-2008-1.pdf.

[50] (2012). MED (Europe in Mediterranean), = ABACUS, A Port Area Energy Consumption and C02 Footprint Calculation Tool. https://www.programmemed.eu/uploads/tx_ausybibliomed/CLIMEPORT_7_ECO_ABACUS_User_manual_EN.pdf.

[51] MED (Europe in Mediterranean) (2011). Methodology Assessment Handbook, CLIMEPORT Project, Program co Financed by the European Regional Development Fund. https://www.programmemed.eu/uploads/tx_ausybibliomed/CLIMEPORT_2_Component_3_-_Executive_Report_EN.pdf.

[52] Port of Rotterdam Authority (2013). CO_2_ Footprint Port of Rotterdam Authority. https://www.portofrotterdam.com/sites/default/files/downloads/co2_footprint_en_factsheet2017.pdf.

[53] Port of Rotterdam (2017). Port of Rotterdam CO_2_ Neutral. https://www.portofrotterdam.com/sites/default/files/port-of-rotterdam-co2-neutral.pdf.

[54] Port of Stockholm (2017). Annual Report and Sustainability Report. https://www.stockholmshamnar.se/siteassets/trycksaker/ports_of_stockholm_2017.pdf.

[55] Port of Gothenburg (2018). Sustainability Report of Gothenburg Port Authority 2018 Sustainable Port. https://www.portofgothenburg.com/FileDownload/?contentReferenceID=14781.

[56] Port of Barcelona (2013). Climate Strategy. http://www.portdebarcelona.cat/en/web/el-port/113.

[57] Ports de la Generalitat (2018). DECLARACIÓ AMBIENTAL. http://ports.gencat.cat/wp-content/uploads/2019/07/Declaracio_ambiental_2018_def.pdf.

[58] Port of San Diego (2013). Climate Action Plan. https://pantheonstorage.blob.core.windows.net/environment/Port-of-San-Diego-Climate-Action-Plan.pdf.

[59] Misra A., Panchabikesan K., Gowrishankar S.K., Ayyasamy E., Ramalingam V. (2017). GHG emission accounting and mitigation strategies to reduce the carbon footprint in conventional port activities—A case of the Port of Chennai. Carbon Manag..

[60] Puerto de Ferrol (2017). Autoridad Portuaria de Ferrol San Cibrao, Memoria Anual 2016. https://www.apfsc.com/wp-content/uploads/2017/07/desempeno-ambiental_2016.pdf.

[61] Tucher M., Stirbu S. (2018). Giurgiulesti International Free Port Report on Carbon Footprint 2017. http://gifp.md/en/wp-content/files_mf/1529738875CarbonFootprintReport2018.pdf.

[62] Tsai Y.T., Liang C.J., Huang K.H., Hung K.H., Jheng C.W., Liang J.J. (2018). Self-management of greenhouse gas and air pollutant emissions in Taichung Port, Taiwan. Transp. Res. Part D Transp. Environ..

[63] Port of Olympia (2018). Port of Olympia 2017 GHG Emissions Inventory Report. https://www.portolympia.com/DocumentCenter/View/3109/2017-Port-of-Olympia-GHG-Emissions-Inventory?bidId=.

[64] Van Duin J.H.R., Geerlings H. (2011). Estimating CO_2_ footprints of container terminal port-operations. Int. J. Sustain. Dev. Plan..

[65] Chowhan S., Hiremath A.M., Asolekar S.R. (2012). Carbon Footprinting of Container Terminal Ports in Mumbai. https://www.researchgate.net/publication/269702576_Carbon_Footprinting_of_Container_Terminal_Ports_in_Mumbai.

[66] Yang Y.C. (2017). Operating strategies of CO_2_ reduction for a container terminal based on carbon footprint perspective. J. Clean. Prod..

[67] Chang Y., Song Y., Roh Y. (2013). Assessing greenhouse gas emissions from port vessel operations at the Port of Incheon. Transp. Res..

[68] Winnes H., Styhre L., Fridell E. (2015). Reducing GHG emissions from ships in port areas. Res. Transp. Bus. Manag..

[69] Williamson T., Hulskotte J., German R., Kirsten M. (2017). Port of London Emissions Inventory 2016, Report to Port of London Authority and Transport for London. https://www.pla.co.uk/assets/finalplaportwideinventoryoutputsreportv10.2publication.pdf.

[70] Olukanni D.O., Esu C.O. (2018). Estimating greenhouse gas emissions from port vessel operations at the Lagos and Tin Can ports of Nigeria. Cogent Eng..

